# Intergenerational differences in dietary acculturation among Ghanaian immigrants living in New York City: a qualitative study

**DOI:** 10.1017/jns.2021.69

**Published:** 2021-09-24

**Authors:** Margrethe F. Horlyck-Romanovsky, Terry T.-K. Huang, Ramatu Ahmed, Sandra E. Echeverria, Katarzyna Wyka, May May Leung, Anne E. Sumner, Melissa Fuster

**Affiliations:** 1 Department of Health and Nutrition Sciences, Brooklyn College, City University of New York, Brooklyn, NY, USA; Center for Systems and Community Design, New York, NY, USA; 2 Department of Health Policy and Management, Graduate School of Public Health and Health Policy, City University of New York, New York, NY, USA; Center for Systems and Community Design, New York, NY, USA; 3 African Life Center, Bronx, NY, USA; 4 Department of Public Health Education, The University of North Carolina at Greensboro, Greensboro, NC, USA; 5 Department of Epidemiology and Biostatistics, Graduate School of Public Health and Health Policy, City University of New York, New York, NY, USA; 6 Nutrition Program, Hunter College, City University of New York, New York, NY, USA; 7 Section on Ethnicity and Health, National Institute of Diabetes and Digestive and Kidney Diseases, and the National Institute of Minority Health and Health Disparities, National Institutes of Health, Bethesda, MD, USA; 8 Tulane University School of Public Health and Tropical Medicine, New Orleans, LA, USA; Center for Systems and Community Design, New York, NY, USA

**Keywords:** Diabetes risk, Dietary acculturation, Generational differences, Ghanaian immigrants, Nutrition transition

## Abstract

Dietary acculturation may explain the increasing risk of diet-related diseases among African immigrants in the United States (US). We interviewed twenty-five Ghanaian immigrants (Youth *n* 13, Age (Mean ± sd) 20 y ± 5⋅4, Parents (*n* 6) and Grandparents (*n* 6) age 58⋅7 ± 9⋅7) living in New York City (NYC) to (a) understand how cultural practices and the acculturation experience influence dietary patterns of Ghanaian immigrants and (b) identify intergenerational differences in dietary acculturation among Ghanaian youth, parents and grandparents. Dietary acculturation began in Ghana, continued in NYC and was perceived as a positive process. At the interpersonal level, parents encouraged youth to embrace school lunch and foods outside the home. In contrast, parents preferred home-cooked Ghanaian meals, yet busy schedules limited time for cooking and shared meals. At the community level, greater purchasing power in NYC led to increased calories, and youth welcomed individual choice as schools and fast food exposed them to new foods. Global forces facilitated nutrition transition in Ghana as fast and packaged foods became omnipresent in urban settings. Adults sought to maintain cultural foodways while facilitating dietary acculturation for youth. Both traditional and global diets evolved as youth and adults adopted new food and healthy social norms in the US.

## Introduction

Dietary acculturation among sub-Saharan African immigrants living in the United States may explain the increased risk of chronic diet-related diseases, including type 2 diabetes (diabetes) and cardiovascular disease, associated with living in the United States in this population^(1–7)^. Dietary acculturation includes changes in eating habits, food procurement habits, as well as cultural practices around cooking and eating, and is associated with increased intake of energy, fat, sugar, sodium and animal protein, and eating more restaurant/take-out meals^(8–10)^. In the US, dietary acculturation and health have been mostly studied among Hispanic/Latino immigrant adults^(11–16)^. Limited research suggests that a lower level of acculturation associated with a healthier diet appears to be more enduring among adult African immigrants than in other immigrant groups, but that African youth experience a higher level of acculturation than adults^(17–20)^.

Understanding how diet across the lifespan affect the risk of non-communicable diseases among African immigrants necessitates an intergenerational perspective. Findings among immigrants from African countries suggest that youth and adults experience immigration and acculturation differently^(20–23)^. Adolescence is a critical time for identity formation where immigrant youth may be exposed to conflicting cultural norms and experience significant challenges^(24,25)^. Furthermore, as child and adolescent immigration to the United States is projected to increase by 30 % by 2040^(26–29)^, examining the effects of acculturation on identity formation and health risks is critically important to anticipate future health needs. Overall, there is a paucity of data on the intergenerational variation in dietary acculturation among African immigrant populations in the United States. To our knowledge, only three studies have explored the adult acculturation experience of African immigrants in New York City (NYC) and included the adult perspective on youth experiences^(21–23)^; none has interviewed three generations within the same immigrant group.

Ghanaians were selected for the present study because they are the largest group of African immigrants to NYC (estimated population 27 400)^(26)^. Ghanaian immigrants tend to belong to transnational families^(30)^. Transnational families are defined as maintaining kinship networks and family ties across borders, e.g. Ghana and the United States, while members live in more than one country^(31)^. Previous dietary acculturation literature has primarily focused on the adult experience with limited exploration of intergenerational dynamics and the effect of food environments and transnationalism on acculturation experiences by age group^(20)^.

The present study aims to address this research gap by adopting an intergenerational approach to (a) understand how cultural practices and the acculturation experience influence dietary patterns of Ghanaian immigrants and (b) identify the intergenerational differences in dietary acculturation among Ghanaian youth, parents and grandparents.

## Materials and methods

### Participant recruitment

Participants were recruited through a variety of methods including the distribution of flyers on college campuses, community organisations and local businesses in African cultural enclaves throughout NYC. Materials were also distributed via social media and personal outreach, including community contacts of the research team. The project focused on careful recruitment and quality of conversation as well as care, time and dignity in analysis^(32)^. Based on prior research protocols examining intergenerational relationships in immigrant families interviewing between 12 and 20^(21,33–35)^ participants, it was anticipated that a level of saturation representing considerable breadth and depth of understanding would be reached with 20–25 people representing eligible families/households.

Participants met the inclusion criteria if they self-identified as Black; currently living in NYC and were of Ghanaian heritage. In addition, each participant had to be a member of a Ghanaian family where one or more members had immigrated to the United States from Ghana and had at least one family member between the age of 13 and 27 years. A screening survey was administered online, in person or over the phone. Participants were chosen through purposeful sampling based on their role as youth, parent or grandparent in a Ghanaian family.

### Data collection

Interviews were held at different community locations (e.g. restaurants, public libraries and mosques). Trained research team members administered consent forms, intake survey and incentives, and co-facilitated interviews. Child assent in addition to parental consent was obtained for participants <18 years of age. Informed consent was obtained for adults ≥18 years of age. Participants completed a brief survey (Supplementary Table S1) before the interviews with questions on demographics, immigration history, health and nutrition behaviour, modelled from the NYC Community Health Survey instrument to mirror quantitative analysis in a larger study^(1,36)^.

To maintain sensitivity to race-related dynamics, the study team included a Black Ghanaian community organiser and five research associates who self-identified as Black and of Jamaican, Trinidadian, Guyanese or Haitian origin in addition to the first author who identified as white and Danish-American. Racial and cultural discordance is openly acknowledged in qualitative research by practising self-reflection and asking culturally relevant and explicit interview questions, thereby increasing the research and participants’ ability to unearth differences and potentially identify topics that may go unexplored if both were of the same background^(37–39)^.

The first author, trained in qualitative methods, facilitated all interviews. One or more research associates were present to facilitate consent, intake questionnaires and note-taking. To prevent response bias by generational group, youth were interviewed in groups or individually and separately from adults. Parents and grandparents were interviewed either together in groups or individually based on participant availability. To protect privacy and confidentiality of the participants, the study did not collect information about relations to other participants. This approach focuses on the perspectives of generational groups rather than the intergenerational perspectives from a few family units.

The semi-structured individual or group interviews followed an interview guide with open-ended questions (Online Resource 2) and were digitally recorded. Between 20 December 2016 and 12 February 2017, we conducted four group interviews (average *n* 3, min 2/max 5) and five individual interviews. Individual interviews lasted on average 50 min and group interviews lasted on average 53 min. All participants received a $20 gift card to a local or online retailer of their choice.

This study was conducted according to the guidelines laid down in the Declaration of Helsinki and all procedures involving human subjects/patients were approved by the City University of New York, Human Research Protection Program, Protocol #2016-1201. Written informed consent was obtained from all subjects.

### Data analysis

Intake survey data were deidentified and summarised to characterise generational groups. All recordings were deidentified and transcribed verbatim by research staff or professional transcription agencies. The research team generated a codebook utilising Grounded Theory Methodology, a complex, dynamic and iterative process in which data collection and analysis alternate^(40)^. Transcribed interviews were coded and analysed with Dedoose version 7.5, using *in vivo* codes, i.e. actual words or expressions of participants to label concepts. The first author and a second team member coded each interview separately and coding was compared iteratively until consensus was reached^(40)^. Emerging themes were included in subsequent interviews. Saturation was reached when no new data emerged from additional interviews and a rich understanding of each research question was reached^(40,41)^. To further elucidate the cultural contexts and intergenerational dynamics that give rise to the unique dietary and health profiles of Ghanaian immigrants, we organised the dietary acculturation themes in a socio-ecological model. The socio-ecological model examines the multiple levels of influence (intrapersonal, interpersonal, organisational, community, and public policy and global forces) with the understanding that behaviours both contribute to and are shaped by these environments.

## Results

### Participant characteristics

Table 1 provides selected characteristics of participants. We interviewed twenty-five Ghanaian New Yorkers; thirteen youth, twelve parents (*n* 6) and grandparents (*n* 6). Parents and grandparents were combined as there was overlap in age, socio-demographic characteristics and qualitative findings. The mean age was 20 ± 5 years among youth and 58⋅7 ± 9⋅7 years among parents and grandparents. Most participants were female (eight youth, six parents and five grandparents). Youth were either enrolled in high school or college or having recently graduated from either. Parents and grandparents were more likely to have a high school education or less. Among those who reported their income, parents and grandparents were more likely to have incomes of ≤$39 000. Most youth did not know their household income.

**Table 1.** Socio-demographic characteristics: Ghanaian youth, parents and grandparents

	Total	Youth	Parents and Grandparents
Population (*n*)	25	52 % (13)	48 % (12)
Mean Age (years ± sd)	38 ± 21⋅2	20 ± 5⋅4	58⋅7 ± 9⋅7
Sex			
Female	75 % (19)	62 % (8)	92 %
Education			
High School or less	68 % (17)	62 % (8)	75 % (9)
Some college or more	24 % (6)	38 % (5)	8 % (1)
Other, incl. vocational school	8 % (2)	0 % (0)	17 % (2)
Income			
0–$39 999	52 % (13)	23 % (3)	83 %(10)
≥$40k	12 % (3)	15 % (2)	8 % (1)
Do not know/Prefer not to answer	36 % (9)	62 % (8)	8 % (1)
Marital Status			
Married/Living with partner	36 % (9)	23 % (3)	50 % (6)
Widowed/Divorced/Separated/Never Married	48 % (12)	62 % (8)	33 % (4)
Prefer not to answer	16 % (4)	15 % (2)	17 % (2)

The family units of the participants included parents and one or few children, or grandparents with adult children and grandchildren living in NYC and Ghana. For most youth, their grandparents were living in Ghana or had died. Participants described family units in NYC, which included both formal and informal family relations formed in the cultural enclave. For example, women would speak of ‘their children’ which included biological children in NYC and Ghana, nieces, nephews and the children of other families in the NYC community. Throughout this project, we came to think of family as individuals who are connected through a web of kinship bonds by blood, legal or informal relations.

Most youth participants (84 %, or eleven out of thirteen) had lived in Ghana with grandparents or other relatives until they came to live in the United States between the ages of 12 and 25 years. Two youth participants had grown up in the US. Parents and grandparents had all lived in Ghana until adulthood, and most had lived in NYC for one or more decades.

### Qualitative findings

We present the qualitative findings by organising themes within the domains of a socio-ecological framework: intrapersonal, interpersonal, organisational, community, and public policy and global forces (Table 2). Each theme was further classified by whether it was reported by either youth or adults or both (indicating convergence). To further clarify the significance of the themes in dietary acculturation, we indicated whether a theme was a force for maintenance of cultural foodways or a force for change. Themes were discussed using a transnational framework, encompassing both experiences in the United States and in Ghana.

**Table 2.** Ghanaian youth, parents and grandparents in New York City (NYC): socio-ecological model domains, place and themes as forces for dietary maintenance and change

Place	Theme	Youth	Convergence	Parents and Grandparents
**Intrapersonal Level**				
New York	‘*We eat the same food*’^a^		*Maintenance*	
Ghana	Growing up in Ghana means preference for Ghanaian food		*Maintenance*	
New York	Preferences for rice over starchy fruits, tubers and roots	*Change*		*Maintenance*
New York	Youth who grow up in the United States rejected eating African food ‘*all the time*’ and developed American food preferences	*Change*		
New York	‘*Everybody wants to have a taste of whatever they like*’	*Change*		
New York	Foods higher in flavour, fat, salt, sugar and calories as more desirable	*Change*		
**Interpersonal Level**				
Ghana	‘*Back in Africa you didn't have a choice … Whatever was put in front of you, that's what you ate*’		*Maintenance*	
New York to Ghana	Parents would send barrels with ultra-processed and packaged foods high in fat, sugar and calories and financial remittances to children still living in Ghana	*Change*		
New York to Ghana	Monetary remittances provided purchasing power	*Change*		
New York	Mothers would cook African foods because they want to teach them ‘*what our food is*’		*Maintenance*	
New York	Youth may reject African foods to fit into United States culture	*Change*		
New York	Mothers would cook some ‘American’ foods for youth	*Change*		
New York	Eating together and from one bowl, but, children, men and women eat separately		*Maintenance*	
New York	Social norms about hand hygiene affect social eating practices out of one bowl	*Change*		*Maintenance*
New York	Busy schedules change meal patterns		*Change*	
New York	‘*Anyone can eat any time they want, anywhere they want’*		*Change*	
New York	More full meals per day	*Change*		
**Organisational Level**				
New York	Nutrition and health education		*Change*	
New York	School lunch is free in NYC, so parents encourage youth to try it	*Change*		
New York	Elders and youth are trying to eat healthier to protect from diet-related diseases		*Change*	
Ghana and New York	Global recognition of chronic disease risk means that healthier eating habits are becoming socially desirable		*Change*	
New York	Documentaries and TV personalities promote healthier lifestyles		*Change*	
New York	Healthier diets after graduating college	*Change*		
**Community Level**				
New York	Food in NYC is aplenty and may lead to larger portion sizes and more frequent meals		*Change*	
Ghana	Foods in Ghana are fresh, clean, organic, and produced without hormones, fertilizers, pesticides and genetically modified organisms		*Maintenance*	
New York	American foods were considered unclean, genetically modified and produced with hormones, fertilizers and pesticides		*Maintenance*	
New York	African food ingredients are expensive		*Change*	
New York	Ingredients to cook African foods are available in NYC Supermarkets	*Maintenance*		
New York	Processed and packaged African foods are not ‘*real’*			*Change*
New York	Concerned about personal hygiene of the person preparing the food	*Maintenance*		
New York	Lots of unhealthy snack options in the cultural enclave	*Change*		
New York	Ubiquitous and affordable ‘fried rice’	*Change*		
New York	Eating out at Ghanaian or African restaurants was equivalent to eating at home and considered an unnecessary expense		*Maintenance*	
New York	Eating out only for special occasions: ‘*Anything that wasn't our food’*		*Change*	
**Public Policy and Global Forces Level**				
Ghana	Media influences and product placement in films and online media		*Change*	
Ghana	Vacations and visits to Ghana expose to Ghanaian community, foods and items brought back to NYC		*Maintenance*	
Ghana	Vacations and visits to the United States expose to American foods		*Change*	
Ghana	Cities have more processed foods, but rural areas get to eat healthier		*Maintenance and Change*	
Ghana	Progress and a move towards achieving higher living standards		*Change*	
Ghana	Globalisation brought fast food, ultra-processed and packaged foods		*Change*	

a Italicized text in quotation marks indicates in vivo codes

#### Intrapersonal level

Youth, parents and grandparents all agreed that ‘we eat the same foods’ and that everyone ate traditional home-cooked Ghanaian foods. The general Ghanaian diet consists of starchy roots (cassava, yams), starchy fruit (plantain) and cereals (maize, rice and wheat) which supply most of the daily energy^(42,43)^. Legumes, tree and ground nuts, dried and smoked fish, beef, goat, lamb and chicken are served in accompanying sauces and soups. In addition, the traditional Ghanaian diet includes leafy greens, an assortment of vegetables (e.g. okra, tomatoes, peppers, gourds and carrots) and a wide variety of fruits. Cooking oils include plant oils (e.g. palm kernel, groundnut, coconut, maize, shea butter or olive). Additional foods include store-bought white bread, cake, pasta and cookies. It is important to note that there are significant regional and cultural variations within Ghanaian foods.

*Growing up in Ghana meant a preference for home-cooked Ghanaian food and regular meals*. Parents noted that although their children may have been born in the US, many were sent to Ghana to be raised by relatives and ensure cultural continuity with a deep appreciation for their cultural heritage, language and foods.

While sharing the same foods in the home was a force of maintenance in NYC, there were small but significant differences between what youth, parents and grandparents ate, in what amounts, how often and when.

*When my son drinks a tea, sometimes I have a taste of the tea just to see how much sugar. See, if the sugar is too much, I talk to him, ‘hey be careful, it's too much for you.’ When he eats rice, when I found out it's too oily, I complain about it.*

Grandfather

Ghanaian youth had clear *preferences for rice over starchy fruits, tubers and roots* and the traditional fufu (e.g. pounded yams, plantains and cassava) or fermented starchy foods such as kenkey. The fondness of rice extended to other cultural cuisines, and rice was seen as a common ground by youth and adults, whether as part of Chinese, Mexican or Jamaican cuisine. In contrast, starchy foods and fufu remained staple comfort foods and forces of maintenance among adults.

*Youth who grew up in the United States or arrived at a young age rejected eating African food* ‘*all the time*’ *and developed American food preferences*. American foods included things like chicken nuggets, pizza, chicken wings, macaroni, and cheese, hamburgers and cold cereals. Youth often expressed that they liked ‘some’ traditional Ghanaian foods but certainly not all and not every day. Foods that parents and grandparents considered comfort foods such as boiled yams, green plantains and fufu were typically not on the list of favourites among youth. In fact, one young Ghanaian woman who grew up in the United States noted:

There were certain things that I *did* like. Plantain, I think it's universal. I love it when [my mom] makes [sweet, fried plantain], not the hard one, the yellow one, I like the sweet taste.

Youth, Female

Two young women also noted that their mothers seemed to accommodate the differences in dietary preferences of each family member.

*[My mother] would cook predominantly Ghanaian food for them [my parents] to eat but she would make us American food*.

Youth, Female

A universal theme was that *everybody wants to have a taste of whatever they like*. Youth sought a greater variety of foods as they moved to NYC and parents accommodated this desire both at home and outside. The variety and affordability of food options available in the United States were desirable by youth and juxtaposed with the absence of choice in Ghana.

*In America, there are different varieties of food and everybody wants to have a taste of whatever they like. [ … ] Here, we can buy Domino's … Pizza … McDonald's, like the $3 burger … Chinese rice.*

Youth, Male

When asked about dietary habits, fruit and vegetable consumption was relatively low for all respondents as 80 % of respondents ate less than two servings the day before the interview. Youth consumed one or more sugar-sweetened beverages per day, whereas none of the parents and only one grandparent reported drinking sugar-sweetened beverages.

Youth also experienced a force for change as they found *foods higher in flavour, fat, salt, sugar and calories as more desirable* than adults. One Ghanaian grandfather who was diagnosed with diabetes and hypertension had identified this difference and felt compelled to convey the healthy eating practices recommended by his doctors.

*That's the most disturbing thing. Because right now, we [are] protecting ourselves from [diet-related diseases], the young generation they are not into that. So, I have to educate [my son] and let him understand the consequences of too much sugar.*

Grandfather

#### Interpersonal level

The notion of choice, as it related to diet, appeared only in the conversations about eating in NYC. *Back in Africa you didn't have a choice … Whatever was put in front of you, that's what you ate …* Youth and adults were clear that living in Ghana meant eating Ghanaian food and whatever was provided.

Youth who grew up in transnational families had unique exposures to American food culture as *parents would send parcels or barrels with ultra-processed and packaged foods high in fat, sugar and calories and financial remittances to children still living in Ghana*. For parents and children who were separated by migration, barrels represented an expression of caring. Despite geographic separation, youth were given access to coveted consumer goods and packaged foods. Barrels as forces for change allowed the adults to extend their financial stability and the United States experience to their children while they were living in Ghana.

*I wanted them to become familiar with American foods because I had the ability to.*

Grandfather

Remittances were important links between Ghana and NYC. The financial stability of the relative living in the United States was often passed on to the family in Ghana. *Monetary remittances provided purchasing power* and served as forces for change as they enabled families in Ghana to purchase necessities as well as more expensive American products, e.g. cereals and cookies.

*I'm trying to keep them happy at home. So, I sent the money to allow them to have all this stuff that will keep kids home. I send the money to tell them to go buy ‘that that that that that’ [Cornflakes and other packaged foods] … so I take safe care of them. So, when they came here, they do for their own children what their daddy did for them.*

Grandfather

Moreover, barrels and financial remittances demonstrated how the dietary acculturation experiences were injected into daily life in the home country by parents, even before youth were able to migrate to the US. Furthermore, these dietary practices and cherished food memories extended to second and third-generation Ghanaians in the US.

For many families living in NYC, *mothers would cook African foods because they want to teach their children* ‘*what our food is*’. Girls would learn to cook starting at the age of 7 or 8. Some boys said that their mothers had taught them to cook, but for most this was limited to cooking rice. Adult men, who had arrived in NYC ahead of their families, taught themselves to cook African foods because they missed the flavours and found that African restaurants were too expensive.

In contrast, *youth* who grew up in NYC *may reject African foods to fit into United States culture*.

*I don't really like African food. So, if [my mother] was making Fufu, I'd be like, ‘can I get some chicken wings? I don't want this Fufu’.*

Youth, Female

From the youth perspective, mothers seemed to accommodate changing food preferences in their children and therefore *would cook some* ‘*American*’ *foods for youth* while still preparing traditional foods for the adults in the family.

*I think she automatically assumed before we grew up, that we wouldn't like it. I really don't know. At some point I wanted cheese, and I didn't love fufu.*

Youth, Female

Youth who grew up in Ghana shared in detail how the children in Ghanaian households may be *eating together and from one bowl, but children, men and women ate separately*. Eating with one hand from a shared dish required skill and dexterity. This form of meal socialisation was a force for maintaining cultural foodways and meal interactions. However, after arriving in the US, *social norms about hand hygiene affected social eating practices out of one bowl*. Adults seemed unphased by these social norms and ate with their right hands at home and in public in the community. Youth noted that, in NYC, the fear of spreading disease by touching each other's food was a force for change and made youth use utensils and individual plates both at home and outside the home.

*Busy schedules also changed meal patterns*. In NYC, food shopping and cooking took place on weekends, and leftovers made up daily meals during the week. The practice of eating meals together was often abandoned in NYC, in part because family members were home at different times of the day. Even in families where members were home at the same time, meals were consumed separately whenever each person was hungry and/or had time to eat.

*But now, anyone can eat any time they want, anywhere they want.*

Youth, Female

The resulting physical and emotional distance from others were forces for change, transforming communal and shared meal experiences into more isolated meal experiences. Meal patterns were also affected by forces for change in several other ways. While living in Ghana, participants had been used to fewer daily meals and snacks primarily because food was made available at home and fewer meals were consumed outside the home. In NYC, youth came to expect *more full meals per day* in addition to snacks and beverages as foods were more readily available inside and outside the NYC home.

One grandfather regretted how his children and grandchildren did not wait for food to be prepared, and instead ate what he did not consider real food (e.g. cornflakes with cold milk and packaged baked goods). In contrast, parents and grandparents preferred cooked food and would wait for the food to be prepared.

*It's not easy, all the time you gonna stand in the kitchen cooking for them. They have the corn flakes, they have all these juices, cereal, they go for it!*

Grandfather

Interestingly, the foods this grandfather listed as taking away from eating home-cooked food in NYC were the very same foods he used to provide for his children as they were growing up in Ghana.

#### Organisational level

Across generations, participants noted an increased awareness of the connection between food and the community's experience with weight gain, hypertension and diabetes. Two-thirds of youth were normal weight (67 %) while parents and grandparents were either overweight (25 %) or obese (75 %). One parent and one grandparent had been diagnosed with diabetes. Risk of diet-related diseases was associated with institutional forces for change when local hospitals provided diabetes/hypertension screenings and health education events that led to increased knowledge about healthy diets. Adults also spoke about the importance of receiving free comprehensive care at two health clinics serving the African immigrant communities. Nutrition and health education classes offered in faith-based organisations, schools and community settings by the local health department and cooperative extension were also forces for change. These experiences led to fears that traditional foods were detrimental to health because of the high calorie and starch content combined with sedentary behaviour associated with living in NYC. *Elders and youth* noted that they *are trying to eat healthier to protect from diet-related diseases*.

*We have a lot of programs here [which] teach about moderation. So, if the youth know what their health is all about then [they] can also reduce the rate of diabetes in the community. They are breaking the cycle now.*

Mother

At the institutional level, schools were a key sphere for divergence between youth and the older generations experiences. This influence was different in Ghana compared with NYC. In Ghana, school lunch was a force of maintenance as it may have been a home-cooked hot meal maintaining cultural practices.

In contrast, *school breakfast and lunch are free in NYC, so parents would encourage youth to try it*. This active encouragement to consume a multicultural ‘American’ menu was a daily force of change which was both practically and financially motivated. It was perhaps also a conscious effort to acculturate youth to the new country of residence and its common foods.

*My father said that I should try it, maybe I'll like it. I did, and now I eat school lunch.*

Youth, Male

Youth also recognised that food experiences at school were a force for change.

*When you go to school and you have pasta shells, taco day, you have all these things so that's [ … ] what you eat. So, that's my palate, your palate wants hamburgers because that's what they're feeding you in school.*

Youth, Female

Going away for college was a force for change noted by parents and grandparents as their children or grandchildren appeared to have far *healthier diets after graduating college*. In fact, two young women who had gone away for college and had returned to living in the city confirmed that although they still would enjoy their mother's homemade African food on occasion, their everyday diet differed from those of their parents and the Ghanaian community. They would travel to other neighbourhoods to purchase ‘healthier’ and ‘better’ products and noted that fruits, vegetables and organic products were more desirable at major retailers outside the cultural enclave such as big box stores and organic markets.

A *global recognition of chronic disease risk means that healthier eating habits are becoming socially desirable*. The longer the duration of residence in the US, the more likely participants were to focus on consuming more fruits and vegetables and engaging in physical activity. Similarly, longer duration of stay increased the number of modifications to traditional foods and decreased the frequency of cooking specific cultural foods.

*My parents have been here for a long time. I don't want to say we don't make Ghanaian food. But they've found a way to make Ghanaian food with American products.*

Youth, Female

Youth noted that for parents who had lived in the United States for a long time, *documentaries and TV personalities promoting healthier lifestyles* were forces for change. These adults had adopted healthier habits such as exercising, juicing, eating more fruits and vegetables and less starchy foods. For youth who had grown up in the US, they relied more on YouTube videos to be informed about healthy lifestyles, but the social norms and dietary trends were similar.

#### Community level

Across generations, participants converged in their perception of the NYC food environment being one where food is *aplenty* and affordable.

*… there are so many things I can afford when I work hard, the liberty, the freedom, and the food and the chicken!*

Mother

*[The food], it's cheaper, it's not different, it's cheaper.*

Mother

Participants noted that the lower cost of fresh ingredients for home-cooked meals was a force for maintenance as it allowed for the preparation of cultural foods. However, it was simultaneously seen as a force for change as it led to increased amounts of food prepared and *may lead to larger portion sizes and more frequent meals*.

Youth who had migrated to the United States within the last 5 years indicated that their meal providers in Ghana were still relying on open-air markets to buy fresh food daily. Youth and parents emphasised that although food was relatively more expensive in Ghana it was cleaner, fresher and healthier. They agreed that *foods in Ghana are fresh, organic, and produced without hormones, fertilizers, pesticides and genetically modified organisms*.

In contrast, despite the affordability of food ingredients in NYC, Ghanaians of all ages expressed concern about the safety and healthfulness of American foods. *American foods were considered unclean, likely genetically modified and produced with hormones, fertilizers and pesticides*.

*When you go to the store you see tomatoes that last for weeks. Tomatoes are not supposed to last like that. They inject whatever they put in it and it just lasts. That's not good.*

Youth, Male

In NYC, the cultural enclave includes African markets and restaurants serving authentic Ghanaian food. However, adults agreed that authentic *African food ingredients are expensive* and that the higher cost was a force for change because it meant that some cultural delicacies were only served on rare occasions.

Youth who had grown up in the United States noted that ingredients to cook African foods are available in NYC supermarkets and multicultural communities throughout NYC. Those who had lived outside NYC knew that this was a relatively isolated phenomenon found in cities with concentrated ethnic communities. It may also be an indication of the immigrant cook's ability to find substitutions for cultural foods in regular supermarkets.

*So, they now have powdered Fufu now. You don't have to go and fetch plantain and cassava. [ … ] There is nothing really that you couldn't get from the supermarket.*

Youth, Female

In contrast, parents and grandparents noted that some of the *processed and packaged African foods are not* ‘*real*’ and that staple replacements, such as boxed instant powdered fufu, were unhealthy and lacked flavour and nutrients. Although life in NYC normalised the use of these instant products, parents found that they were unwelcome forces for change and inferior to homemade fufu. In addition, they were concerned that instant fufu was responsible for the weight gains associated with moving to NYC.

Concerns about the healthfulness of American foods were mostly applied to foods found in supermarkets, and few connected these concerns to prepared foods. When a group of Ghanaian youth was asked whether they worried about genetically modified ingredients in pizzas from a popular franchise, they all agreed that they were only *concerned about personal hygiene of the person preparing the food*:

*No, I'm not even thinking about them when I eat Domino's. [ … ] I want to make sure the person that made my food washed his hands.*

Youth, Male

Moving to NYC meant that youth gained independence. Due to busy work schedules of parents, and the availability of food in the urban setting, youth would be given money to buy food after school. They identified *unhealthy* but inexpensive and socially desirable *snack options in the cultural enclave* such as fast food, chips and soda which they had not been consuming as regularly in Ghana but were able to easily buy in NYC. Youth agreed that they ate fast food more often than their parents and grandparents, although many knew that their parents disapproved.

*[My father] doesn't like eating that food. If he sees us, he starts lecturing us like, ‘You shouldn't be eating this food, it's not healthy, it's not good for us, we could get fat.’ So, we just listen to him and we stop eating.*

Youth, Male

One global food consumed outside the home was the *ubiquitous and affordable* ‘*fried rice*’ available from take-out restaurants in many NYC neighbourhoods. Considered a snack, youth would eat it more often than adults.

*I eat [fried rice] once in three months; my kids eat it all the time.*

Mother

Eating at *Ghanaian or other African restaurants in NYC was equivalent to eating at home and considered an unnecessary expense*. This was particularly noteworthy because several of the participants’ family members either owned or worked in African restaurants.

*We didn't teach them to go out and eat, we always make sure we have something at home and eat. Only once in a while we go out because we have to save money. We don't want to be homeless.*

Mother

When asked about *eating out for special occasions* such as birthdays and anniversaries, youth participants would instead mention going to restaurants serving *anything that wasn't our food* such as American, Italian, Chinese or ‘buffet-style’ restaurants.

*We would go to eat out if it was somebody's birthday, like birthdays were big [ … ] Cheesecake Factory, anything that wasn't our food. We still do that when we're all together.*

Youth, Female

#### Public policy and global forces level

Establishing food habits in Ghana and the United States happened in the larger context of globalisation and the nutrition transition. *Globalisation brought fast food, ultra-processed and packaged foods* and sugar-sweetened beverages which may have been forces for change for Ghanaian youth during high school and early adulthood. Moreover, *media influences and product placement* of unhealthy fast food and processed foods *in films and online media* in Ghana was noted by both youth and adults as forces of change prior to moving to the US. Younger generations were more likely to notice and seek these products. Adults were more likely to purchase them for their children upon request, but not for their own consumption.

Global forces also influenced consumption by facilitating the movement of people. While living in Ghana, *vacations and visits to the United States would expose them to American foods*. Youth spoke fondly about the American foods they would eat during visits to the United States and bring back to Ghana as welcomed forces of change. At the same time, *vacations and visits to Ghana exposed to foods and items brought back to NYC*. Both youth and adult participants spoke of frequent travel between the United States and Ghana for business and family reunions. As forces of maintenance, they identified the traditional meals they would be served in Ghana as well as the Ghanaian foods brought back to NYC.

Youth noted that in Ghana, supermarkets as global forces of change are an urban phenomenon. Rural residents in Ghana may have healthier diets than urban residents only because of limited access to highly processed foods. However, they also explained that changing social norms that promote healthy eating may be a conscious choice for some urban residents despite the proliferation of highly processed foods. This may be a manifestation of the nutrition transition progressing beyond the highly processed foods, possibly reviving rural foodways and adopting healthier eating habits among the most privileged.

*Because the cities have more processed foods and the villages don't have that, so they get to eat healthier. The people from the city eat healthy as a choice.*

Youth, Female

The presence of packaged and ready-to-eat foods in Ghana was welcomed, and brand name consumption was seen by some as *progress and a move towards achieving higher living standards*.

## Discussion

To our knowledge, we are presenting the first study to examine, contrast and compare the generational differences in dietary acculturation experiences among the largest Africa-origin group of Ghanaian immigrants living in NYC. The present study offers a new perspective on dietary acculturation in which youth, parents and grandparents all actively seek and facilitate dietary change, but in diverse ways. Previous research posits that dietary acculturation is a dynamic and non-linear exchange in which elements of two or more cultures merge^(10,44)^. Our findings demonstrate that the merging of multiple cultures is actively initiated and facilitated by both youth and adults. Second, our analysis showed that dietary acculturation begins in the home country, continues as the migrants move between Ghana and the US, and evolves as immigrants adopt emerging and changing social norms around food and health in the United States and globally.

Using a socio-ecological and transnational framework, the present work demonstrates that interpersonal factors were part of maintenance of transnational linkages between the United States and Ghana. In transnational families, barrels with food as well as financial remittances are key drivers for cultural change and constitute perhaps the most significant remote acculturative tools yielded by family members. The economic significance of remittances to families in Ghana also should not be underestimated both individually and collectively. In 2018, the value of annual remittances (electronic transfers and goods primarily from the US, Canada and Great Britain) to Ghana was estimated at US$3⋅8 billion or 7⋅3 % of Ghana's GDP^(45,46)^. This economic influx facilitates access to new foods, further accelerating the dietary acculturation process before migration, however, it also contributes to physiological stress experienced by caregivers separated from their children^(47)^.

Transnational linkages are also forged through global forces and the media dissemination of food advertising and US-based food norms. Brand loyalty plays a significant role in dietary acculturation particularly among youth in emerging economies^(48–50)^. Global food corporations, such as Nestlé Group, Mondelez International and Unilever, have accumulated diverse portfolios of global brands^(51)^ and used innovative distribution methods such as direct-sales models transform food systems in low- and middle-income markets for decades^(51,52)^. Products such as Milo^®^ chocolate milk powder and Maggi^®^ bouillon cubes are synonymous with West African food culture^(53,54)^. These global forces may be further reinforced by the care packages sent by family members in the US. Nevertheless, parents and grandparents seemed relatively unaffected by the global trends for their own consumption but often sought to provide access to global food items for youth.

The presence of global products in Ghana, care packages from family members, availability in urban supermarkets and mass media marketing contribute to what has been identified as remote acculturation^(55,56)^, acknowledging that dietary changes no longer begin at the point of entry into the United States or Europe, but commences prior to immigration^(16,17,57)^. Although these effects may be less prominent in Ghana than other regions of the world such as the Caribbean and Central America^(16,56,58)^, North American and European food products have long been perceived as more desirable and of higher status than local foods in Ghana^(59,60)^.

Globalisation and the ability to import food items from the home region also contribute to the recreation of foodways and cultural continuity in the US. African markets carry some speciality foods from Ghana or the West African region. In addition, animal protein sources such as freshly slaughtered chicken or goat are readily available in many NYC neighbourhoods. The availability and affordability of ingredients to prepare Ghanaian foods in NYC may lead to more frequent and increased consumption. Known as the ‘festival food syndrome’, foods that were once only eaten on special occasions in the home country appear more regularly in the immigrant diet, potentially increasing the risk of diet-related disease^(61)^.

The present study also uncovered conflicting perceptions of whether Ghanaian food is healthy or not. Intake data showed that Ghanaians of all ages ate very few servings of fruits and vegetables. However, our conversations made it clear that fruits and vegetables are integral parts of everyday Ghanaian meals. Participants answered dietary survey questions literally as fruit and vegetables served separately and did not account for cooked vegetables consumed as part of soups and stews. In addition, participants perceived starchy vegetables such as yams, plantains and cassava as unhealthy starchy foods not included in fruits and vegetables. Such incongruence in dietary assessment reveals several critical issues in dietary acculturation research. First, United States nutrition assessment methods fail to fully appreciate and accurately reflect what Ghanaian immigrants eat. Such ‘othering’ of cultural identities and foods is a rejection of both the foods and the people^(62)^, and contributes to unreliable reporting of food consumption. Second, dietary assessment tools are based on Euro-normative meal traditions, assuming that foods are served as separate components on individual plates. This precludes adequate assessment of eating practices that do not fall within such narrow definitions of meals, e.g. communal eating from a shared plate^(63)^. As such, dietary assessments and evaluations of dietary change in the Ghanaian community are inadequate. This presents a unique opportunity for public health professionals to collaborate with the Ghanaian community to develop novel and culturally inclusive nutrition and health assessment methods.

As Ghanaian families reunite in the US, youth experience rapid dietary acculturation as they are encouraged to experience and consume more American foods through school lunches, snacks and fast food restaurants. In contrast, adults are far more established in their food preferences and cultural identities, as a product of the amount of time lived in the culture of origin^(64–66)^. The food environment in the cultural enclave, with its mixture of African food stores, cultural institutions and African restaurants, is perceived differently by youth and adults. Youth are more likely to seek food and eating experiences that differ from their culture of origin, such as pizza, fast food and fried rice. In contrast, adults see the enclave as rich in familiar resources and known foods and ingredients. Thus, the cultural enclave simultaneously exposes youth to the acculturative forces for change whereas forces for maintenance protect adults by enabling them to cook and eat authentic Ghanaian food both at home and outside the home. Among youth, frequent consumption of fast food and beverages in addition to eating home-cooked food at home may lead to over-consumption and increased risk of chronic disease. However, these behaviours appear only among adolescent youth and may be attenuated with age. Ghanaian youth who have attended college in the United States indicate dietary and health behaviours supportive of lower chronic disease risk. In addition, living outside the cultural enclave also seems to expose to more diverse food environments and social norms around healthier foods.

Although the Ghanaian community maintains a healthy immigrant advantage and experience lower risk of chronic disease than the US-born Black population^(7)^, youth and adults are acutely aware that living in the United States increases their risk of chronic diseases. Parents and grandparents are adopting and promoting a diet richer in plant foods, in part by re-incorporating healthier foods from ‘back home’ and by decreasing intake of unhealthy foods. Such health awareness is likely influenced by health and nutrition education offered through schools, faith-based and healthcare institutions in NYC. These developments represent a later stage of the nutrition transition^(67)^. In the nutrition transition, the world's emerging economies are rapidly progressing from receding famine and food insecurity to an increased risk of obesity and chronic disease. Our work among Ghanaians illustrates that they may progress equally rapidly towards behaviour changes that promote health and prevent diet-related disease as social norms change globally^(68,69)^. This presents unique opportunities to tailor culturally appropriate nutrition and food education campaigns in NYC which elevate and celebrate the healthfulness of fresh, clean foods that are aligned with Ghanaian food cultures of origin.

### Limitations

The present study generated important insights into the intergenerational differences in dietary acculturation, but some limitations should be noted. To protect privacy and confidentiality we did not collect information about kinship. Therefore, we were unable to identify any similarities or differences between youth, parents and grandparents from the same families or households. In addition, our definition of a family as a group of persons united by the ties of marriage, blood or adoption, did not account for the transcultural family models including formal and informal kinships found in the Ghanaian community in New York City. Therefore, we interviewed members of the community as they self-identified as youth, parent or grandparent in a Ghanaian immigrant family. Furthermore, we acknowledge a significant overlap between the parent and grandparent generations, where several participants identified as both parents and grandparents because they had both children and grandchildren between the ages of 13 and 27. Therefore, our findings were not unique for the two older groups and the groups were combined. The parent and grandparent sample was comprised mostly of women. In our recruitment, efforts women were more likely to enrol in our study than men. Arguably, this is an issue in most food and nutrition research. Given that women tend to be in charge of food activities, they may be more inclined to participate in this type of research. While gender representation is important, it was not the main focus of our work, requiring a different recruitment approach to fully capture gender diversity. A future study may be best equipped to examine gender differences. In addition, Ghanaian men cited limited time, limited benefit and fear of deportation. Our research interviews were conducted between December 2016 and March 2017, during which time presidential executive orders barring immigration from African countries were issued. Thus, the community was justifiably concerned about sharing personal information.

We interviewed members of the Ghanaian community in NYC and gathered important insights into the intergenerational variation in dietary acculturation and chronic disease risk in this community. The qualitative approach of our study does not provide for generalisation of findings to all Ghanaian immigrants. Ghana includes a rich diversity in terms of regional, cultural, ethnic and religious affiliation that our research was unable to capture. Future quantitative studies may build on our findings to examine generational differences while capturing the diversity of the community within the US context.

Despite these limitations, the present research contributes a more detailed understanding of the intergenerational differences and similarities in dietary acculturation experience among Ghanaian immigrants. These findings generate important hypotheses to be tested in future quantitative studies.

## Conclusion

The present study demonstrates that for Ghanaian immigrants, dietary acculturation begins at the interpersonal level in the home country and is perceived as a positive process. It is an active and deliberate progression by which adults provide socially desirable food for their children as made possible by increased income, access and social mobility. The dietary acculturation process differs significantly by generation, where youth appear to embrace the unhealthy foods available outside the home, and parents and grandparents are more likely to prefer home-cooked meals and outside meals aligned with the family's food culture of origin. Nevertheless, increasing awareness of the contribution of dietary factors and sedentary lifestyles to the risk of obesity, hypertension and diabetes may motivate adults to focus on weight loss and consumption of more fruits and vegetables, less starch and smaller portions overall.

As access and affordability of healthier foods improve in urban settings in the United States, healthy food practices will also become part of the forces for change along the acculturation continuum. Ghanaian immigrants of all ages living in NYC concurrently seek to maintain cultural foodways, embrace dietary acculturation and attempt to adopt healthier eating habits to prevent chronic disease. These findings present unique opportunities to facilitate active lifestyles and healthier food environments in cultural enclaves through participatory urban planning and intentional design; to foster improved health across the lifespan among Ghanaian immigrants in New York City.

## Supplementary material

For supplementary material accompanying this paper visit http://dx.doi.org/10.1017/jns.2021.69. click here to view supplementary material
