# Supplementary material for: Intergenerational differences in dietary acculturation among Ghanaian immigrants living in New York City: a qualitative study
Source: J Nutr Sci. 2021 Sep 24;10:e80. doi: 10.1017/jns.2021.69 (PMC8477345; doi:10.1017/jns.2021.69)
Supplement: Supplementary file 1 [file S2048679021000690sup001.docx]

Supplementary Table 1S. Intake Questions

| **(Answer choices) or [Fill in the blank]** |
| --- |
| 1. Please indicate your unique 12-digit identifying number (The research team will provide you with this) [___] |
| 1. What is your gender? (Male; Female; Other; Prefer not to answer) |
| 1. What is your age? [___] |
| 1. What is you race/ethnicity? (Please select all that apply.): (American Indian/Alaska Native; Asian / Pacific Islander; Black/ African American; Hispanic / Latino; White / Caucasian; Prefer not to answer.) |
| 1. What is the highest level of education you have complete or the highest degree you have received? (Elementary / Primary school; Middle/ Secondary (Lower/Upper) school; Less than high school or equivalent (e.g. GED) or A-levels; Some college but no degree; Associate degree; Bachelor degree; Master degree; Doctoral degree; Other; Prefer not to answer) |
| 1. Would you say in general that your health is: (Excellent; Very Good; Good; Fair; Poor; Don’t know; Prefer not to answer) |
| 1. About how tall are you without shoes? (For example, if you are 5 feet and 4 inches, write 5'4") [___] |
| 1. About how much do you weigh without shoes? (Please indicate pounds or kilograms) [___] |
| 1. How often do you drink sugar sweetened beverages? This would include soda, juice, sweetened ice tea, fruit punch, sports drinks, sweetened coffee, or tea. (Never; Less than one per day; One per day; More than one per day; Don’t know; Prefer not to answer) |
| 1. On average, how many times per week do you eat meals that were prepared in a restaurant? Please include eat-in restaurants, carry out restaurants and restaurants that deliver food to your house. [___] |
| 1. Thinking about nutrition...how many total servings of fruit and/ vegetables did you eat yesterday? A serving would equal one medium apple, a handful of broccoli, or a cup of carrots. [___] |
| 1. During the past 30 days, other than your regular job, did you participate in any physical activities or exercises such as running, golf, gardening, sports, hiking, swimming, bicycling, or walking for exercise? (Yes; No; Don’t know; Prefer not to answer) |
| 1. Have you ever been told by a doctor, nurse, or other health professional that you have diabetes? (Yes; No; Don’t know; Prefer not to answer) |
| 1. How many people currently live in your household? [___] |
| 1. How many generations live in your household? [___] |
| 1. Where did each of your family members grow up? (Ghana; United States; Somewhere else; Don’t know) |
| 1. Of the family members who were born abroad, at what age did they come to live permanently in the US? (As a child, under age 5; As a child between ages 5 and 18; As an adult, over age 18; Did not immigrate; Don’t know) |
| 1. Of these income groups, which best represents your family income in the last 12 months? (No income; Less than $20,000; $20,00-$39,999; $40,000-59,000; $60,000-$79,999; $80,000-$99,999; $100,000 or more; Don’t know; Prefer not to answer) |
| 1. What best describes your marital status? (Married; Widowed; Divorced; Separated; Never married; Living with a partner; Prefer not to answer) |
| Questions adapted from the New York City Community Health Survey Questionnaire. [Ref] |

Supplementary Table 2S. Interview Script: Questions and Probes

| 1. Tell us a little bit about what being a Ghanaian immigrant family living in New York City means to you. |
| --- |
| 1. When we think of food, we often think of the finished meal, but getting food sometimes means farming and gardening, or it means walking a long or short way to the store, or it may mean eating what you can get from the corner store. If you were to think of your childhood, how did you and your family get food? [allow for multiple family settings/locations]    1. -and when you think of cooking and eating, who cooked it, where did they cook and when?    2. -and when you think of eating, where and when would you eat and with whom? |
| 1. If you think of your family, household, and community now, where do you get food?    1. -and when you think of cooking now, who cooks the food, where and when do they cook?    2. -and when you think of eating meals now, how are they similar and how are they different from the Ghanaian meals in the past? |
| 1. How are the meals of your [INSERT parents / children / grandchildren] similar to or different from your meals?    1. Can you explain why that might be? |
| **Up to now, we have been talking about past and current food practices. This last set of questions pertains to health.** |
| 1. How would you describe your own health? |
| - 1. How would you describe the health of your Ghanaian: Parents, grandparents, children, or grandchildren]? |
| 1. In our research we have found that |
| - 1. The Ghanaian immigrant community may be at lower risk of having diabetes/sugar and being obese than both Black Caribbean immigrants and African Americans |
| - 1. Can you help explain these differences? |
| - 1. How do you think that these health outcomes differ between older generations and younger generations of Ghanaians? |
| 1. What are some of the other health issues that affect your Ghanaian family and community? |
| 1. Why do you think these particular issues affect your community? |
| 1. What is there about Ghanaian food culture, health, and family dynamics that we did not ask but you think we should know about? |
